# Predicting Mechanical Properties of Polymer Materials Using Rate-Dependent Material Models: Finite Element Analysis of Bespoke Upper Limb Orthoses

**DOI:** 10.3390/polym16091220

**Published:** 2024-04-26

**Authors:** Syed Hammad Mian, Usama Umer, Khaja Moiduddin, Hisham Alkhalefah

**Affiliations:** 1 Advanced Manufacturing Institute, King Saud University, Riyadh 11421, Saudi Arabia; 2 King Salman Center for Disability Research, Riyadh 11614, Saudi Arabia

**Keywords:** finite element analysis, Bergström–Boyce model, three network viscoplastic model, upper limb orthosis, customization, 3D printing, mechanical testing

## Abstract

Three-dimensional printing—especially with fused deposition modeling (FDM)—is widely used in the medical field as it enables customization. FDM is versatile owing to the availability of various materials, but selecting the appropriate material for a certain application can be challenging. Understanding materials’ mechanical behaviors, particularly those of polymeric materials, is vital to determining their suitability for a given application. Physical testing with universal testing machines is the most used method for determining the mechanical behaviors of polymers. This method is resource-intensive and requires cylinders for compression testing and unique dumbbell-shaped specimens for tensile testing. Thus, a specialized fixture must be designed to conduct mechanical testing for the customized orthosis, which is costly and time-consuming. Finite element (FE) analysis using an appropriate material model must be performed to identify the mechanical behaviors of a customized shape (e.g., an orthosis). This study analyzed three material models, namely the Bergström–Boyce (BB), three-network (TN), and three-network viscoplastic (TNV) models, to determine the mechanical behaviors of polymer materials for personalized upper limb orthoses and examined three polymer materials: PLA, ABS, and PETG. The models were first calibrated for each material using experimental data. Once the models were calibrated and found to fit the data appropriately, they were employed to examine the customized orthosis’s mechanical behaviors through FE analysis. This approach is innovative in that it predicts the mechanical characteristics of a personalized orthosis by combining theoretical and experimental investigations.

## 1. Introduction

In the current highly competitive business environment, there is an increasing need to enhance, simplify, and expedite design and fabrication processes. Additive manufacturing (AM) or 3D printing is a cost-effective method used to fabricate complex shapes with less waste compared with traditional subtractive manufacturing methods [1]. Among the fabrication techniques, fused filament fabrication (FFF), also known as fused deposition modeling (FDM), is widely used. In this process, a solid material, typically a raw filament, is forced through a heated nozzle at temperatures below the melting point but above the glass transition temperature. Then, this quasi-melted filament is gradually applied to a bed layer by layer to construct the component from the bottom to the top [2]. FDM is widely used owing to its ease of use, accessibility, and cost-effectiveness [3]. This tendency to create the highest quality product while minimizing resource usage is crucial for success across various industries. Thus, computational tools are increasingly being employed at the start of design processes to minimize reliance on trial-and-error methods and physical trials. Conducting physical tests to investigate mechanical properties directly can be expensive and time-consuming, particularly when numerous parameters are involved [4]. Alternative methods involve employing mathematical simulations that utilize numerical techniques. The finite element (FE) method is a well-established technique that enables accurate forecasts of deformations and stresses in products under both normal and accelerated loading conditions [5]. As a result of ongoing advancements in FE processes, multiple commercially accessible FE packages can effectively conduct complex simulations.

Polymers demonstrate both elastic and viscous material properties [6]. The dynamic behavior of 3D-printing-based elastic polymers has garnered significant interest from researchers in the fields of applied physics, material science, and engineering. Given polymeric materials’s key role in the 3D printing industry, advanced material models should be incorporated into simulation tools to achieve accurate analysis and design.

The polymeric materials acrylonitrile butadiene styrene (ABS), polylactic acid (PLA), and polyethylene terephthalate glycol (PETG) are widely employed in medical settings [7,8]. When these polymers are utilized in biomedical devices, such as orthoses’ structural components, it is necessary to include additional factors to accurately describe their mechanical behavior. Furthermore, the mechanical behaviors of polymers are correlated with their thermal properties, becoming particularly significant under a high strain rate [9]. When polymers undergo substantial deformations, they generate heat due to the loss of energy caused by inelastic viscosity. This leads to an increased material temperature, resulting in thermal softening [10]. Thermal effects significantly impact polymers’ mechanical properties under a high strain rate [11]. In addition, material nonlinearity caused by plastic behavior poses a challenge because it introduces a highly complex mathematical formulation in the analysis. Moreover, experimental studies for polymer materials require a substantial amount of time and financial resources [12]. To overcome these issues, numerical analysis is often employed. Moreover, advanced constitutive models in simulation tools should be used for accurate analysis and design.

Mathematical constitutive models provide a framework to elucidate the mechanical behaviors of materials in any given context [13]. These models can vary in complexity and may rely on various factors that typically represent a material’s mechanical properties [14]. To have reliable numerical simulations, it is essential to accurately quantify the parameters (i.e., constants) employed in the governing equations of the chosen constitutive model. Model calibration entails the mechanical characterization of materials. Various constitutive methodologies have been developed to examine polymers’ mechanical characteristics. Initial methodologies, such as those used by Hughes [15], have elucidated the mechanical behaviors of polymers through the application of conventional isotropic, rate-independent plasticity utilizing the Mises yield criterion. Numerous studies have reported the utilization of FE simulations for the analysis of polymer materials for orthosis applications [16,17,18].

Elastomers are essential components in the study of medical devices, including orthoses. Some of these applications subject elastomers to significant deformations, small strain, and dynamic loads to test the material’s nonlinear viscoelastic behavior. Various methodologies are necessary to comprehensively investigate polymeric materials in medical equipment applications. The Bergström–Boyce (BB) model [19,20], three-network (TN) [21,22], and three-network viscoplastic (TNV) models [23,24] are commonly used in Abaqus as a build-in model and are known for their accuracy and utility.

This study examined and evaluated three constitutive mathematical models, namely the BB, TN, and TNV models, to accurately describe the mechanical behaviors of PLA, ABS, and PETG used in individualized upper-limb orthoses. These models have been calibrated for diverse 3D printing materials and could be employed in the field of biomedical engineering. These models can be used to examine orthosis devices, such as upper limb orthosis that are capable of being forced to undergo dynamic loading. Furthermore, these models provide additional opportunities for the examination of deformations and their implications.

## 2. Materials and Methods

Three constitutive models were compared to evaluate the behavior of thermoplastic materials in unpredictable deformation scenarios. Three material models, namely the BB, TN, and TNV implemented in ABAQUS/Standard from Dassault Systems (Dassault Systems, Vélizy-Villacoublay, France), were used to determine their suitability. These models were independently simulated for three polymer materials, namely PLA, ABS, and PETG, to assess their mechanical behavior. The objective was to develop an accurate and validated constitutive material model to select the most appropriate polymer material for hand orthosis.

**Figure 1:**
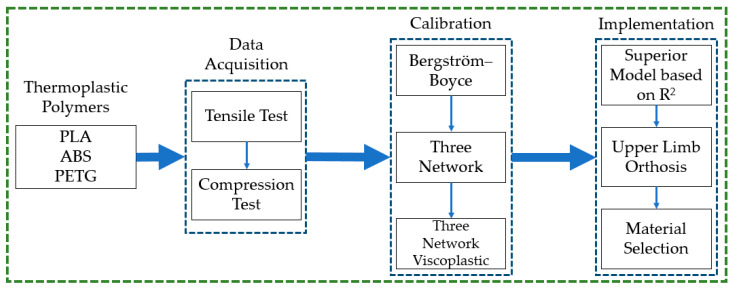
Methodology adopted to identify suitable material for upper-limb orthosis.

Two main components of the proposed approach were used to calibrate the models using data obtained through tensile and compression testings for specific materials, as well as using the calibrated material models, to forecast the mechanical behaviors of the three polymers for hand orthosis. The study methodology is illustrated in Figure 1.

### 2.1. Data Acquisition

PLA, ABS, and PETG were examined in this investigation. In the data-collection phase, the true mechanical behaviors of 3D-printed specimens were explored based on these materials. Three constitutive material models were calibrated using information gathered from tensile and compression tests. These models are covered in detail in the subsequent section. For data acquisition, the first step was to fabricate standard specimens (dog-bone and cylindrical-shaped specimens) using a 3D printer.

**Figure 2:**
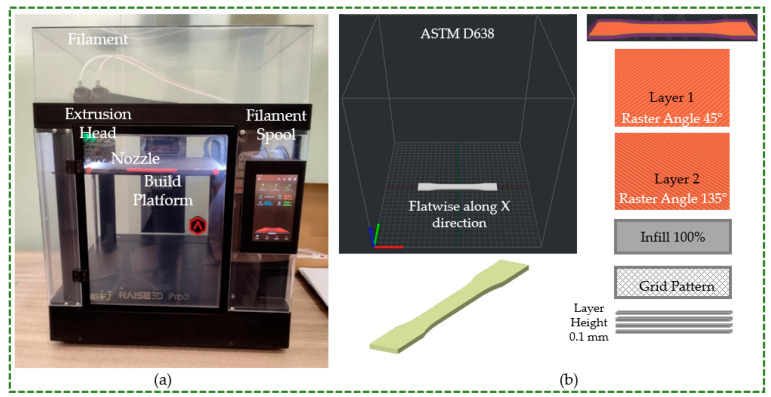
(**a**) Open-filament 3D printer (**b**) Graphical representation of process parameters.

As illustrated in Figure 2a, the specimens employed in this work were produced utilizing an open-filament 3D printer called the Raise 3D Pro 3 (Raise 3D Technologies Inc., Irvine, CA, USA). The specimens were produced using the ideal printing parameters suggested by Raise 3D, the material filament supplier. As demonstrated in Figure 2b, the test specimens were constructed flatwise along the X-direction. The raster angle of the grid infill pattern was altered by 90° between two succeeding layers. For instance, one layer was printed at a raster angle of 45°, followed by a subsequent layer printed at 135°, and so on, until the entire component was produced (refer to Figure 2b). The same layer height of 0.1 mm and infill percentage of 100% were used to produce all of the specimens. All materials have ideal temperature settings and optimum speed, which ensures seamless and precise 3D printing. Consequently, PLA, ABS, and PETG were processed using the Raise-3D-recommended speed and default temperature settings (bed and nozzle temperatures) [25,26,27]. Table 1 lists he 3D printing parameters applied for producing standard specimens using various materials.

Tensile and compression test results were deployed to calibrate the constitutive material models. The mechanical behaviors were examined using tensile and compression tests. Printed samples were subjected to compression and tensile testing based on ASTM standards. The standards used were ASTM D638 [28] and ASTM D695 [29] for tensile and compression tests, respectively.

**Figure 3:**
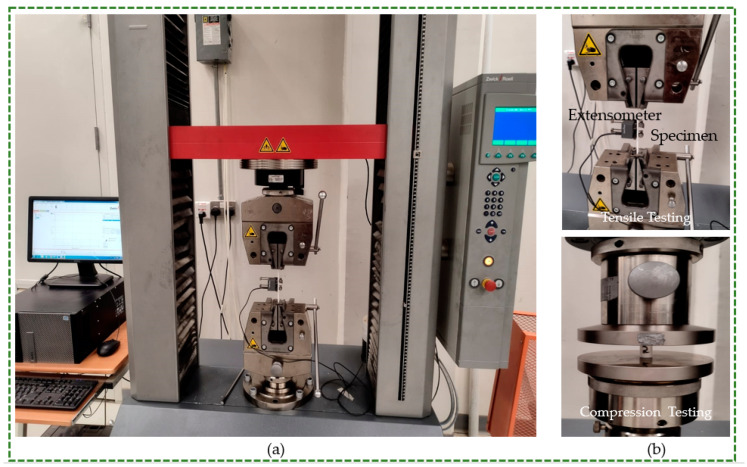
(**a**) Universal testing machine and (**b**) tensile and compression testing setups.

As shown in Figure 3a, tensile and compression tests were performed using a Zwick Z100 electromechanical universal testing machine (ZwickRoell, Ulm, Germany) fitted with a cell with a 100 kN load. In the elastic range, the modulus of elasticity was experimentally measured using an extensometer. To measure elongation, the extensometer was fastened to the tensile test sample (Figure 3b). This enabled an accurate calculation of Young’s modulus [30]. Identifying the yield point, which denotes changes in the material’s behavior from elastic to plastic, is difficult. If yield restriction is crossed, the material is permanently plastically deformed. The 0.2% strain offset approach is useful for determining yield strength [31]. The 0.2% offset (yield strength) is the stress value that equates to 0.2% plastic strain. Thus, this technique was used to calculate yield strength [32].

It is vital to use data for various strain rates when calibrating constitutive material models. Thus, tension and compression samples were deformed with varying rates of displacement control. These tests were conducted at various strain rates. The strain rates for tensile tests were 0.0005 s^−1^ and 0.005 s^−1^, and those for compression tests were 0.018 s^−1^, 0.011 s^−1^, and 0.0011 s^−1^. The stress–strain experimental curves were recorded for each test conducted at room temperature.

### 2.2. Material Models

Material responses were captured using the BB, TN, and TNV constitutive material models. The details of these models are as follows.

#### 2.2.1. BB

The BB is a comprehensive material model used to forecast the large-strain, time-dependent behavior of elastomers [19,33,34,35]. BB as a material model was used to replicate the mechanical behaviors of thermoplastic materials.

The imposed deformation gradient in the BB model acts on F_A_ and F_B_, two parallel macromolecular networks [33]. The deformation gradient operating on network B is broken down into viscoelastic and elastic constituents as F = F*_B_*^e^ F*_B_*^v^. The response of network A is provided by an eight-chain model using Equation (1).

(1) σA=μJλ*¯ L−1λ*¯λLL−11λL dev [b*] + κ (J−1) I

Stress on network B is estimated using the eight-chain model (see Equation (2)), although the effective shear modulus differs.

(2) σB=sμJBeλBe*¯ L−1λBe*¯λLL−11λL  dev [bBe*]+κ (JBe−1)I

where $\lambda_{B}^{e*}$ refers to the chain stretch in the elastic portion of Network B and *s* is a dimensionless material property indicating the shear modulus of Network B in relation to Network A. Equation (3) can be employed to determine the total Cauchy stress.

(3) $$\sigma =\sigma_{A}+\sigma_{B}$$

The velocity gradient on network B *L_B_* can be separated into elastic and viscous elements (refer to Equation (4)).

(4) $$L_{B}=L_{B}^{e}+\tilde{L_{B}^{v}}$$

where $L_{B}^{e}$ = $\dot{F_{B}^{e}}$ ${\left(F_{B}^{e}\right)}^{-1}$; $\tilde{L_{B}^{v}}$ = $\tilde{D_{B}^{v}}+\tilde{W_{B}^{v}}$; $L_{B}^{v}$ = $\dot{F_{B}^{v}}$ ${\left(F_{B}^{v}\right)}^{-1}$ = $D_{B}^{v}$ + $W_{B}^{v}$

$\tilde{W_{B}^{v}}$ ≡ 0 must be specified to make the unloading distinct. Equation (5) is used to constitutively define the viscous deformation rate of network B.

(5) $$\tilde{D_{B}^{v}}=\dot{Y_{B}}\;(\sigma_{B,\;b_{B}^{e*}})\;N_{B}^{v}$$

where $N_{B}^{v}$ = $\frac{dev[\sigma_{B}]}{\tau }$ = $\frac{dev[\sigma_{B}]}{{\left\|dev[\sigma_{B}]\right\|}_{F}}$

τ is the effective stress driving the viscous flow. The time derivative of $\dot{F_{B}^{v}}$ can be expressed using Equation (6).

(6) $$\dot{F_{B\;}^{v}}=\dot{Y_{B}^{v}}{\left(F_{B}^{e}\right)}^{-1}=\frac{dev\;\left[\sigma_{B}\right]}{{\left\|dev\;[\sigma_{B}]\right\|}_{F}}\;F_{B}^{e}\;F_{B}^{v}$$

Equation (7) provides the rate equation for viscous flow.

(7) $$\dot{Y_{B}^{v\;}}=\dot{Y_{o}}{\left({\bar{Y}}_{B}^{v}-1+\xi \right)}^{C}{\left[R(\frac{\tau }{\tau_{base}}-\hat{\tau_{cut}})\right]}^{m}$$

R (x) = (x+x)2 is the ramp function, $\hat{\tau_{cut}}$ represents the cutoff stress beyond which no flow will occur; a constant, $\dot{Y_{o}}$ ≡ 1/s, is included to ensure dimensional continuity, and ${\bar{Y}}_{B}^{v}$ = $\sqrt{\frac{tr\left[b_{B}^{v}\right]}{3}}$ denotes the viscoelastic chain stretch. The expression for effective stress causing the viscous flow is defined by Equation (8).

(8) $$\tau ={\left\|dev\;[\sigma_{B}]\right\|}_{F}=\sqrt{\left[\sigma_{B}^{\prime }\sigma_{B}^{\prime }\right]}$$

#### 2.2.2. TN

Three components, or parallel-acting molecule networks, constitute the TN model, a material model designed especially for thermoplastic materials [22]. Entropic resistance governs the massive strain response in this model, whereas two distinct energy-activation methods equivalent to amorphous and semi-crystalline domains characterize the early viscoplastic response [36].

In this model structure, the deformation gradient operating on network A is broken down into elastic and viscoplastic elements: F = F*_A_*^e^ F*_A_*^v^. An eight-chain model with temperature dependence provides the Cauchy stress applying on network A (refer to Equation (9) [20,37].

(9) σA=μAJAeλAe*¯ 1+θ−θoθ^L−1λAe*¯λLL−11λL  dev [bAe*]+κ (JAe − 1) I

where $J_{A}^{e}$ = det [$F_{A}^{e}$], with $\mu_{A}$ representing the initial shear modulus and *λ_L_* denoting chain locking stretch. The current temperature is represented by θ, while θ_0_ is the reference temperature, and $\hat{\theta }$ is a material parameter that indicates the stiffness’s temperature dependence. The Cauchy–Green deformation tensor is expressed as $b_{A}^{e*}$ = ($J_{A}^{e}$)^−2/3^ $F_{A}^{e}$ ($F_{A}^{e}$)^T^, and ${\bar{\lambda }}_{A}^{e*}$ = $\sqrt{\frac{tr\left[b_{A}^{e*}\right]}{3}}$ indicates the effective chain stretch dependent on the eight-chain topology hypothesis [37],$\;L^{-1}$ (*x*) represents the inverse Langevin function, where L (*x*) = coth (x)−1x and *κ* indicate the bulk modulus.

The viscoelastic deformation gradient for network B can be split into elastic and viscoplastic elements: F = F*_B_*^e^ F*_B_*^v^. By adopting the eight-chain network model as in network A, network B’s Cauchy stress can be calculated using Equation (10).

(10) σB=μBJBeλBe*¯ 1+θ−θoθ^L−1λBe*¯λLL−11λL  dev [bBe*]+κ (JBe − 1) I

where $J_{B}^{e}$ = det [$F_{B}^{e}$], $\mu_{B}$ is the initial shear modulus and $b_{B}^{e*}$ = ($J_{B}^{e}$)^−2/3^ $F_{B}^{e}$ ($F_{B}^{e}$)^T^ defines the Cauchy–Green deformation tensor. The effective chain stretch is represented as ${\bar{\lambda }}_{B}^{e*}$ = $\sqrt{\frac{tr\left[b_{B}^{e*}\right]}{3}}$. With the introduction of plastic strain, the effective shear modulus changes from an original value of *μ_Bi_* to a final value of *μ_Bf_*, as per Equation (11).

(11) $$\dot{\mu_{B}}=-\beta [\mu_{B}-\mu_{\mathrm{Bf}}]\cdot \dot{Y_{A}}$$

where $\dot{Y_{A}}$ represents the viscoplastic flow rate defined in Equation (14). The model captures the transition from the initial yield to large-scale flow by modulating stiffness with plastic strain.

Equation (12) determines network C’s Cauchy stress, adopting the eight-chain model with I_2_-dependence of the first order.

(12) σC=11+qμCJλchain 1+θ−θoθ^L−1λchain¯λLL−11λLdev[b*]+κ(J−1)I+qμCJ [I1*b*−2I2*3I−b*2]

where J = det [F], $\mu_{C}$ is the initial shear modulus, $b^{*}$ = (J)^−2/3^ F (F)^T^ is the Cauchy–Green deformation tensor, $\lambda_{chain}$ = $\sqrt{\frac{tr\left[b^{*}\right]}{3}}$ represents the effective chain stretch, and q modulates the I_2_ dependence.

The system’s total Cauchy stress is expressed by Equation (13).

(13) $$\sigma \;=\sigma_{A}\;+\sigma_{B}\;+\sigma_{C}$$

To complete the description of this model framework, the rate kinematics should be determined. The rate kinematics for network A’s total velocity gradient, L = $\dot{F}F^{-1}$, can be split into elastic and viscous components: *L* = $L_{A}^{e}+{\left(F_{A}^{e}L_{A}^{v}F_{A}^{e}\right)}^{-1}$ = $L_{A}^{e}+\tilde{L_{A}^{v}}$, where $L_{A}^{v}$ = $\dot{F_{A}^{v}}$ ${\left(F_{A}^{v}\right)}^{-1}$ = $D_{A}^{v}$ + $W_{A}^{v}$ and $\tilde{L_{A}^{v}}$ = $\tilde{D_{A}^{v}}+\tilde{W_{A}^{v}}$.

An undefined rigid body rotation of an intermediary state maintains the stress-free state, resulting in the nondistinct characterization of unloading processes coupling the deformed state with the intermediary state. Several techniques can be employed to make the intermediary state distinctive [38], one of which is to specify $\tilde{W_{A}^{v}}$ ≡ 0. Elastic and inelastic deformation gradients incorporating rotation will generally arise from this. After this, network A’s viscoplastic flow rates can be determined utilizing Equation (14).

(14) $$\tilde{D_{A}^{v}}=\dot{Y_{A}}N_{A}$$

The term $\dot{Y_{A}}$ indicates the true deviatoric flow rate, whereas the tensor N_A_ specifies the driving deviatoric stress’s direction, which is transferred from the relaxed to the current configuration.

Given that σ_A_ is calculated in a loaded configuration, the driving deviatoric stress transferred from the relaxed to the current configuration is given as $\sigma_{A}^{\prime }$ = *dev*[*σ_A_*]. The effective stress, denoted by *τ_A_*, is defined using the Frobenius norm *τ_A_* = ${\left\|\sigma_{A}^{\prime }\right\|}_{F}\;$= $\sqrt{tr[\sigma_{A}^{\prime }\sigma_{A}^{\prime }]}$. Thus, the direction of the driving deviatoric stress N*_A_* is $\frac{\sigma_{A}^{\prime }}{\tau_{A}}$. The effective deviatoric flow rate is then determined by the power law described in Equation (15).

(15) $$\dot{Y_{A}}=\dot{Y_{o}}{\left(\frac{\tau_{A}}{\hat{\tau_{A}}+aR(p_{A})}\right)}^{mA}\cdot {\frac{\theta }{\theta_{o}}}^{n}$$

where $\dot{Y_{o}}$ ≡ 1/s is included to guarantee dimensional continuity, $p_{A}$ = −[${\left(\sigma_{A}\right)}_{11}$ + ${\left(\sigma_{A}\right)}_{22}$ + ${\left(\sigma_{A}\right)}_{33}$]/3 indicates hydrostatic pressure, *R*(*x*) = (*x* + |*x*|)/2 represents ramp function, and $\hat{\tau_{A}}$, a, mA, and n are material parameters. This paradigm assumes that the flow rate’s temperature dependence takes the form of power law. To summarize, the viscoelastic flow of the velocity gradient of network A can be determined by applying Equation (16).

(16) $$\dot{F_{A}^{v}}=\dot{Y_{A}^{v}}{\left(F_{A}^{e}\right)}^{-1}=\frac{dev\;[\sigma_{A}]}{\tau_{A}}\;F$$

Network B’s total velocity gradient can be determined as that of network A. For example, L = $\dot{F}F^{-1}$, can be split into elastic and viscous components: *L* = $L_{B}^{e}+{\left(F_{B}^{e}L_{B}^{v}F_{B}^{e}\right)}^{-1}$ = $L_{B}^{e}+\tilde{L_{B}^{v}}$, where $L_{B}^{v}$ = $\dot{F_{B}^{v}}$ ${\left(F_{B}^{v}\right)}^{-1}$ = $D_{B}^{v}$ + $W_{B}^{v}$ and $\tilde{L_{B}^{v}}$ = $\tilde{D_{B}^{v}}+\tilde{W_{B}^{v}}$. The intermediary state is defined by $\tilde{W_{B}^{v}}$≡ 0. Network B’s viscoplastic flow rate is determined utilizing Equation (17).

(17) $$\tilde{D_{B}^{v}}=\dot{Y_{B}}\;N_{B}=\frac{dev[\sigma_{B}]}{\tau_{B}}$$

where τ_B_ = ${\left\|\sigma_{B}^{\prime }\right\|}_{F}$≡ = $\sqrt{tr[\sigma_{B}^{\prime }\sigma_{B}^{\prime }]}$

The deviatoric flow rate can be estimated using Equation (18).

(18) $$\dot{Y_{B}}=\dot{Y_{o}}{\left(\frac{\tau_{B}}{\hat{\tau_{B}}+aR(p_{B})}\right)}^{mB}\cdot {\frac{\theta }{\theta_{o}}}^{n}$$

where $\dot{Y_{o}}$ ≡ 1/s is included to ensure dimensional continuity, $p_{B}$ = −[${\left(\sigma_{B}\right)}_{11}$ + ${\left(\sigma_{B}\right)}_{22}$ + ${\left(\sigma_{B}\right)}_{33}$]/3 indicates hydrostatic pressure, and $\hat{\tau_{B}}$, a, mA, and n are material parameters. The viscoelastic flow of the velocity gradient of network B can be measured using Equation (19).

(19) $$\dot{F_{B}^{v}}=\dot{Y_{B}^{v}}\;{\left(F_{B}^{e}\right)}^{-1}=\frac{dev\;[\sigma_{B}]}{\tau_{B}}\;F$$

#### 2.2.3. TNV

The TNV is a versatile viscoplastic material model that can accurately represent experimentally observed behaviors of numerous thermoplastics, including volumetric plastic flow, damage accumulation, time- and pressure-dependent plastic flow, pressure-dependent bulk modulus, and triaxiality-dependent failure [39]. One-, two-, or three-parallel networks and one additional failure model are used in the TNV model. The material model’s fourth parameter indicates the failure model type, whereas the initial three parameters identify the type of network for each network.

The TNV model is a substitute for the TN one. In many cases, TNV is more accurate and more extensive than TN. Moreover, volumetric plastic flow can be predicted using TNV.

### 2.3. Abaqus Implementation

FE models for tensile testing have been developed using ABAQUS/CAE. Besides ABAQUS, there are other effective numerical techniques such as the finite difference method [40], the bezier multi-step method [41], and the differential quadrature method [42] which can be utilized for numerical analysis. These approaches are unique because they can handle complex geometries, are robust, and can be applied to large-scale engineering simulations. This paper employed ABAQUS for numerical analysis because of the authors’ expertise and the program’s well-established capabilities.

**Figure 4:**
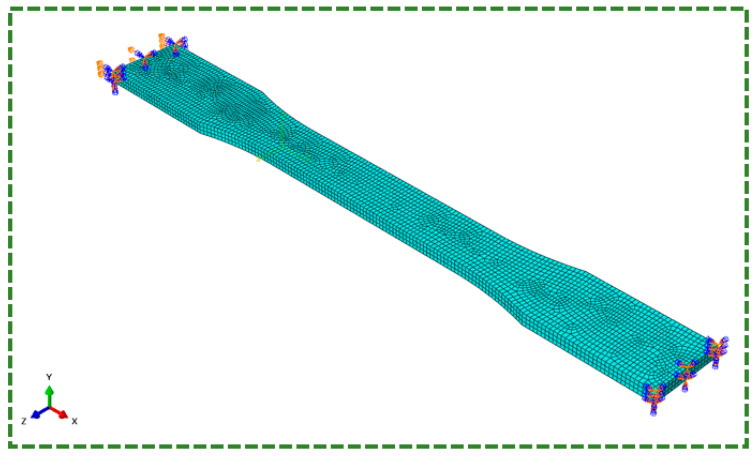
Boundary conditions for simulation in ABAQUS.

The FE model had been discretized using 8,322 C3D8R elements (i.e., eight-node brick elements with decreased integration and hourglass control) in ABAQUS. One end of the specimen was fixed, i.e., all structural degrees of freedom were constrained, whereas the other end had a displacement boundary condition (BC) for the required strain (refer to Figure 4). The Mesh and BCs are shown in Figure 4. The BB, TN, and TNV models were implemented using the PolyUmod plugin for ABAQUS developed by PolymerFEM LLC, for the calculation of stresses at each integration point [43]. Visco analysis was conducted using ABAQUS/STANDARD using direct method and asymmetric matrix storage.

The implementation of the BB, TN, and TNV models in ABAQUS is computationally more challenging than that of other viscoelastic and viscoplastic models. The complete material behavior is decomposed into two or three networks to accurately capture the material nonlinear response at different strain and strain rates. This requires the determination of the deformation gradient and Cauchy stress tensor for parallel and series networks. For example, with the BB model, the true behavior of a certain polymer can be decomposed into two parallel networks (e.g., A and B) with non-linear hyperelastic behavior. In addition, network B also contains non-linear viscoelastic behavior elements connected in series. This requires additional steps for the calculation of a consolidated deformation gradient considering both elastic and viscoelastic components. The accuracy of the models depends on model parameters selection and the number of experimental data available for calibration and testing. For example, using a TN model to predict a typical thermoplastic without considering the temperature effects at least requires both uniaxial tension and compression data at two different strain rates.

## 3. Results and Discussion

**Figure 5:**
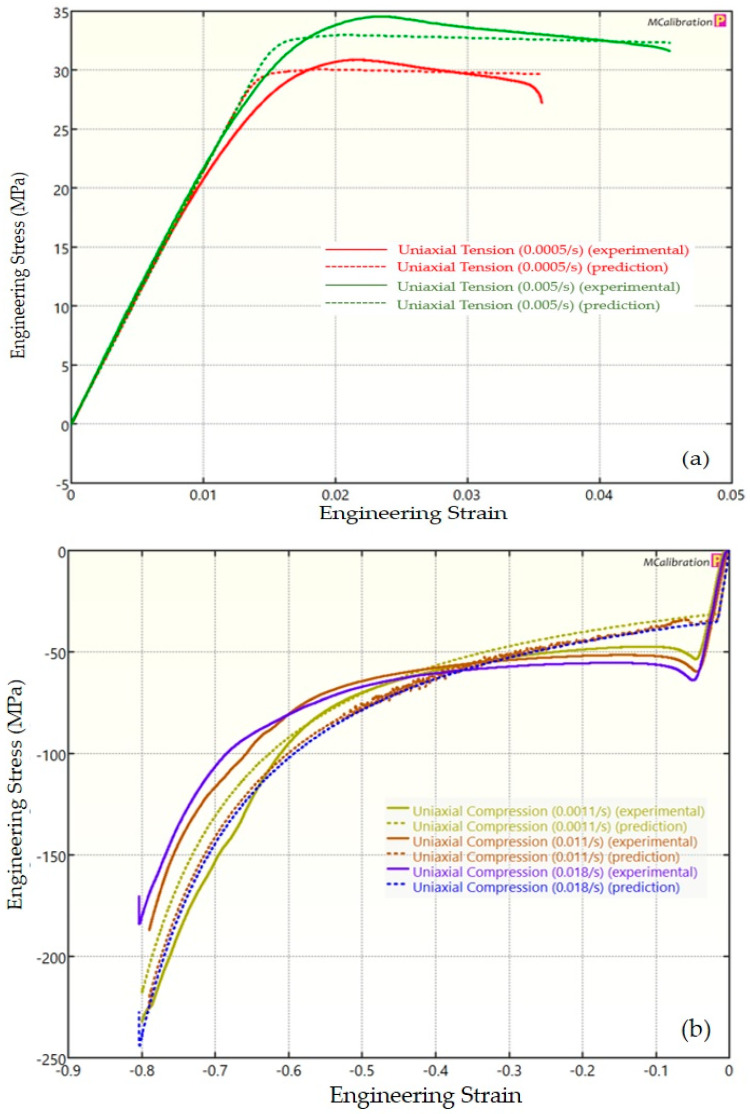
Experimental and BB predicted results for PLA: (**a**) uniaxial tension; (**b**) uniaxial compression.

The experimental findings of uniaxial tensile testing and the numerical results obtained using the BB model for PLA, ABS, and PETG are illustrated in Figure 5a, Figure 8a and Figure 11a, respectively. The data indicated that all three materials exhibited nearly linear elastic behavior initially before beginning to soften and yield until ultimate breakage. This indicates that these materials depend substantially on strain rate. The flow stress was found to be highly sensitive to strain rate. The flow stress increased as the strain rate increased. In the present investigation, it was observed that the tensile strength, yield strength, Young’s modulus, etc., all increased as the strain rate increased for all three polymers. Similar outcomes of increased mechanical characteristics at a greater strain rate have also been reported in the literature [44,45,46,47]. The identical results were observed in the current study, where an increase in strain rates led to an increase in all mechanical properties. This improvement in the mechanical characteristics at greater strain rates can be attributed to the reduced time available for polymer relaxation and rearrangement processes. The shorter test duration inhibits the formation of flaws or cracks and reduces the likelihood of failures due to in-layer or inter-layer fracture modes [46]. There is another justification for greater mechanical properties at higher strain rates. When strain rate rises, strain hardening also increases because the stress-relaxation time decreases. This leads to the formation of additional dislocations and defects in the material, which lowers molecular mobility [48]. In addition to enhanced strength at higher strain rates, a slightly higher elongation at break was also noted at higher strain rates for all three materials. This increase in elongation at break can be a result of thermal softening. At higher strain rates, friction inside the polymer chains causes localized heating, which increases the temperature. This temperature rise causes the polymer to become softer, which increases the elongation before failure. Similar outcomes of increased elasticity modulus, ultimate strength, and total elongation at higher strain rates were reported by Cui et al. [49] and Wang et al. [46] for glass-fiber-reinforced thermoplastic composites. In summary, the self-heating in the current investigation was minimal at the strain rates that were investigated, and the stress–strain curves showed no significant indications of thermal softening. Nonetheless, at strain rates considerably higher than those explored here, the consequences of thermal softening might be substantial.

For PLA, plastic deformation occurred at about 2% strain, resulting in a Young’s modulus of 2.28 GPa, tensile strength of 30.84 MPa, and a breaking strain of 3.56%. Under tensile stress, the ABS curve displayed a region of elastic and plastic deformation that displayed a stress–strain response that was initially linear, similar to PLA. Furthermore, ABS’s elongation at break (3.86%) was only slightly longer than that of PLA. Furthermore, it was found that ABS was 20% weaker than PLA, with a tensile strength of 24.58 MPa. Additionally, it was observed that, up until the ABS sample fractures, the resistive stresses remained nearly constant (following the yield point) with an increase in strain. PETG exhibited the lowest elastic modulus—that is, the gradient of the stress–strain curve in an elastic region—compared with PLA and ABS. The elastic modulus of PETG was measured to be 1.64 GPa. Furthermore, PETG was as strong as PLA, but it became considerably more brittle and did not extend considerably when it reached its yielding point. There is a high chance of failure because PETG does not deform plastically.

A linear zone exists for all the materials (uniaxial compression testing) wherein the material experiences elastic deformation and restores to its initial size upon stress removal (Figure 5b, Figure 8b and Figure 11b). This region ends when a material reaches its yield point and begins to exhibit plastic behavior. In this situation, when stress is released, it no longer reverts to its initial size. However, the linear zone displays varying elongation based on material, indicating that some materials have higher elastic regimes than others. Thus, different compressive properties are anticipated. For instance, at a strain rate of 0.0011/s, PLA exhibited the highest yield compressive strength at 51.93 MPa, followed by ABS at 50.03 MPa and PETG at 44.94 MPa.

The initial step in a material model’s execution is its calibration to the uniaxial experimental data. The best estimate of the material’s properties must be made using the entire set of uniaxial data. These models can be utilized for prediction once the proper material parameters are determined. For the most precise calibration, the automatic extensive procedure was selected. The automatic optimization process continued until the solution could no longer be improved. This approach toggles between various optimization methods to identify the suitable combination of material parameters. This technique involves various algorithms, including random search, the Levenberg–Marquardt algorithm, and NEWUOA search. Table 2, Table 3 and Table 4 for BB, TN, and TNV, respectively, provide the final material properties.

**Figure 6:**
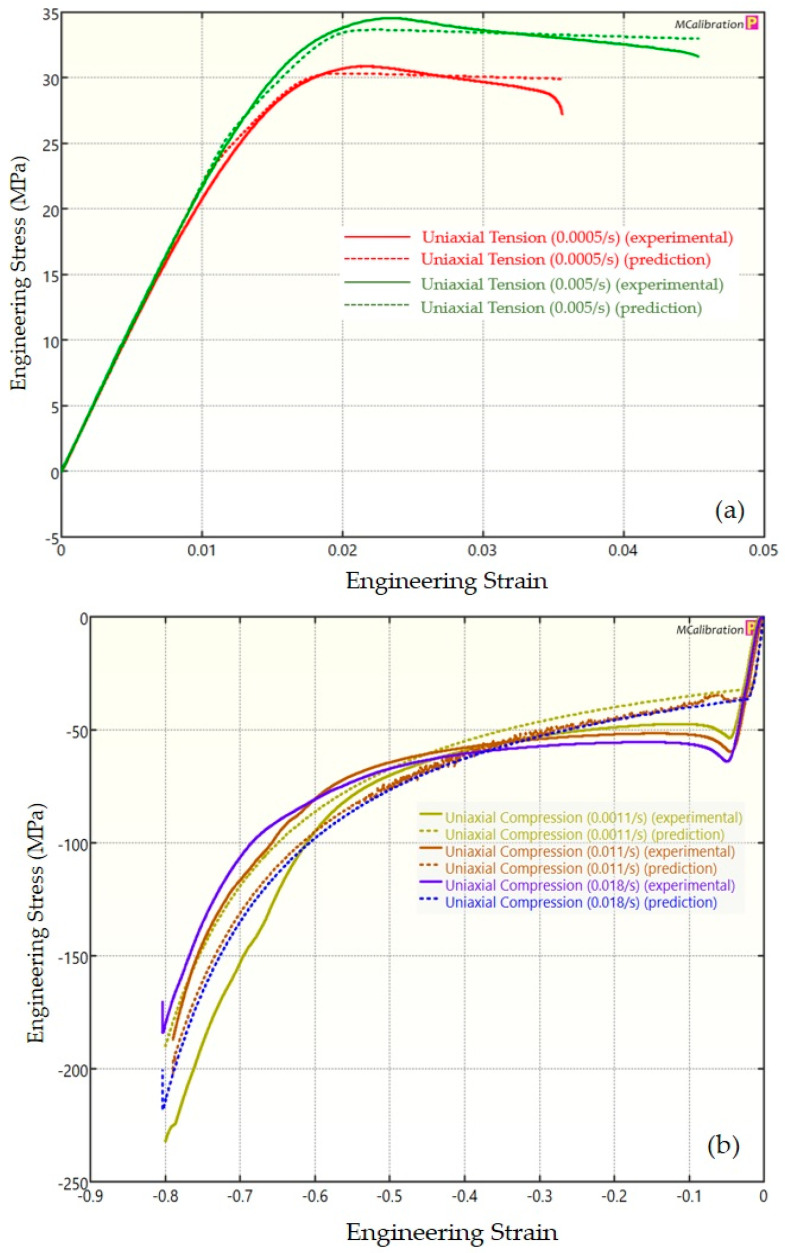
Experimental and TN predicted results for PLA: (**a**) uniaxial tension; (**b**) uniaxial compression.

**Figure 7:**
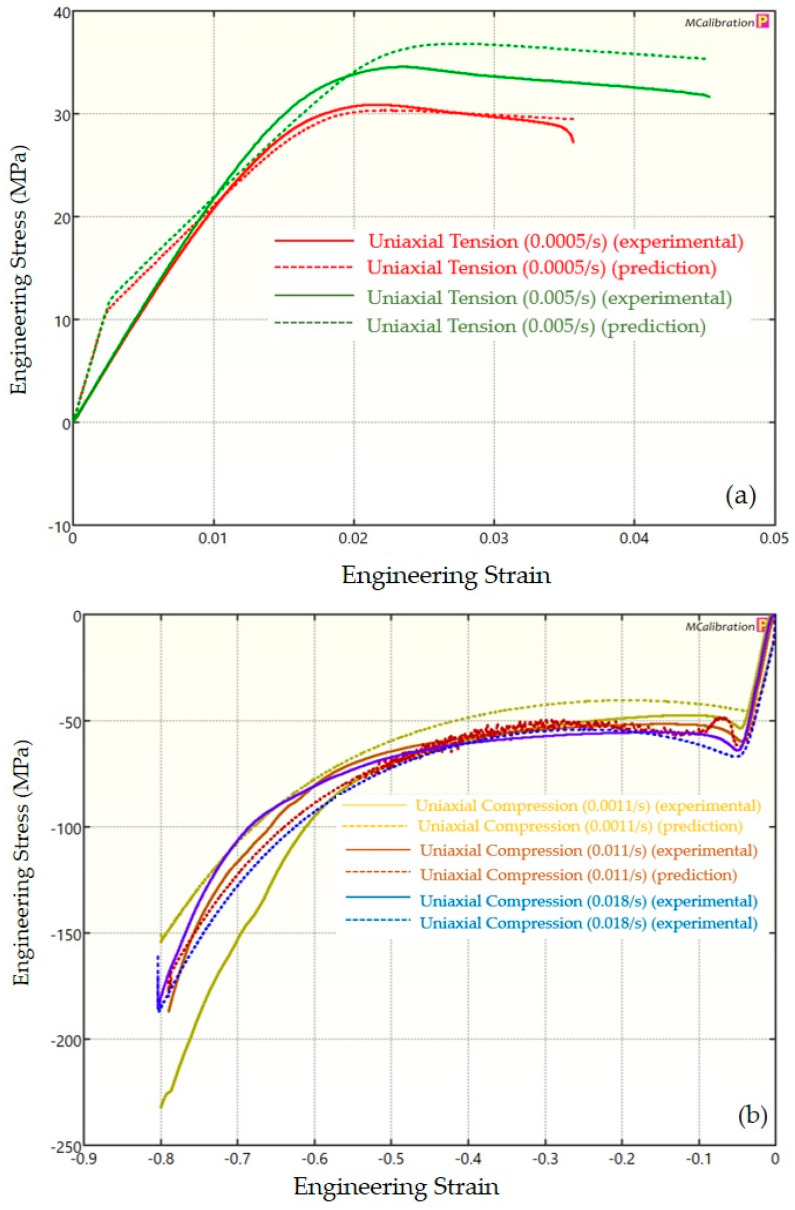
Experimental and TNV predicted results for PLA: (**a**) uniaxial tension; (**b**) uniaxial compression.

**Figure 8:**
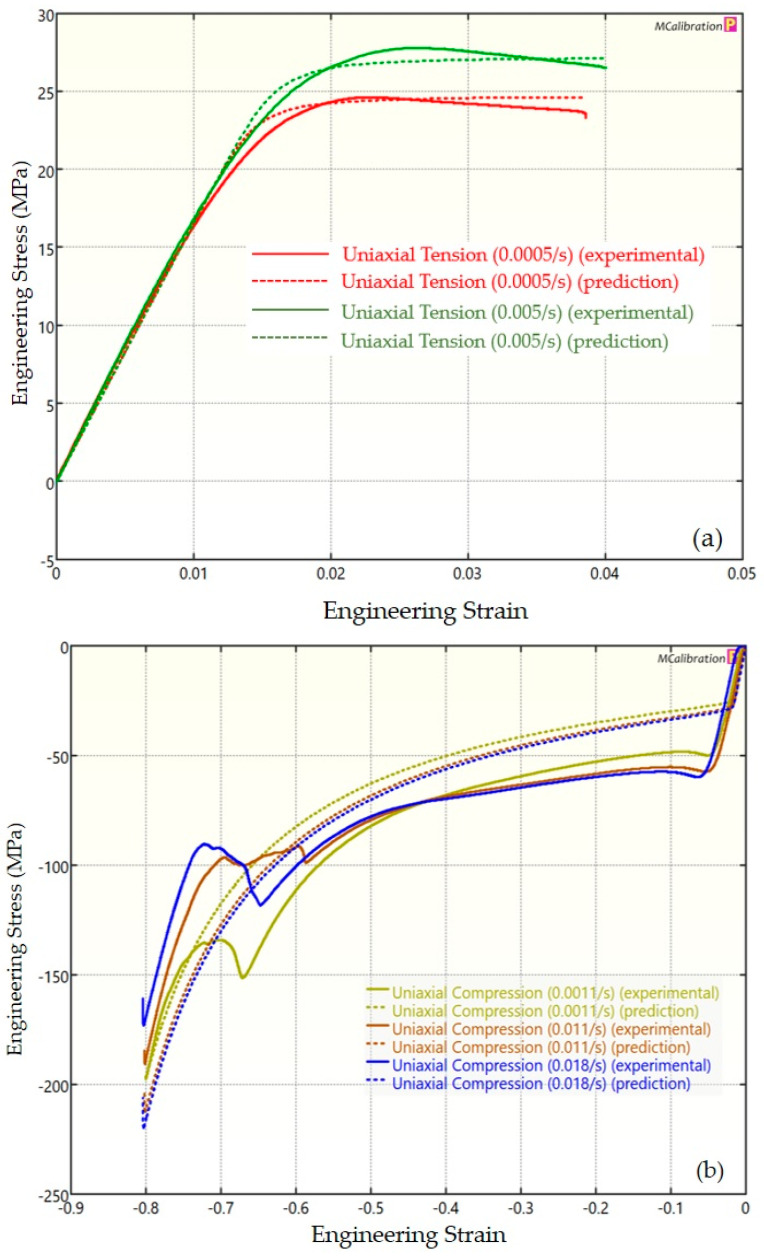
Experimental and BB predicted results for ABS: (**a**) uniaxial tension; (**b**) uniaxial compression.

**Figure 9:**
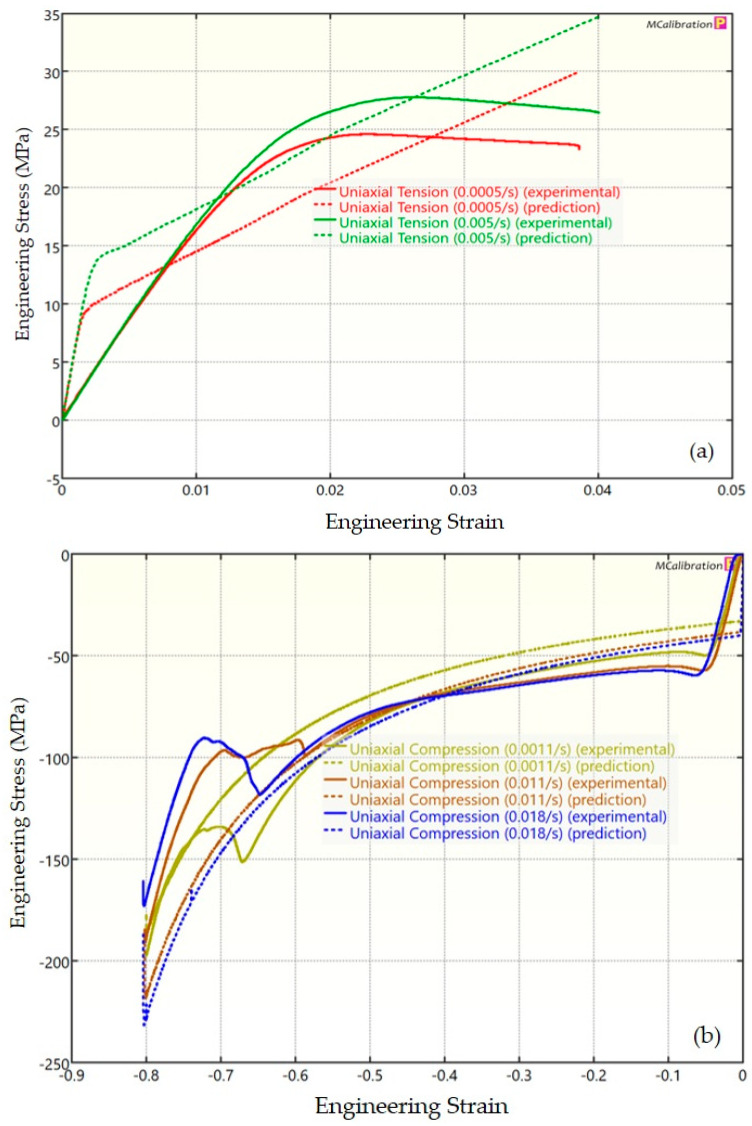
Experimental and TN predicted results for ABS: (**a**) uniaxial tension; (**b**) uniaxial compression.

**Figure 10:**
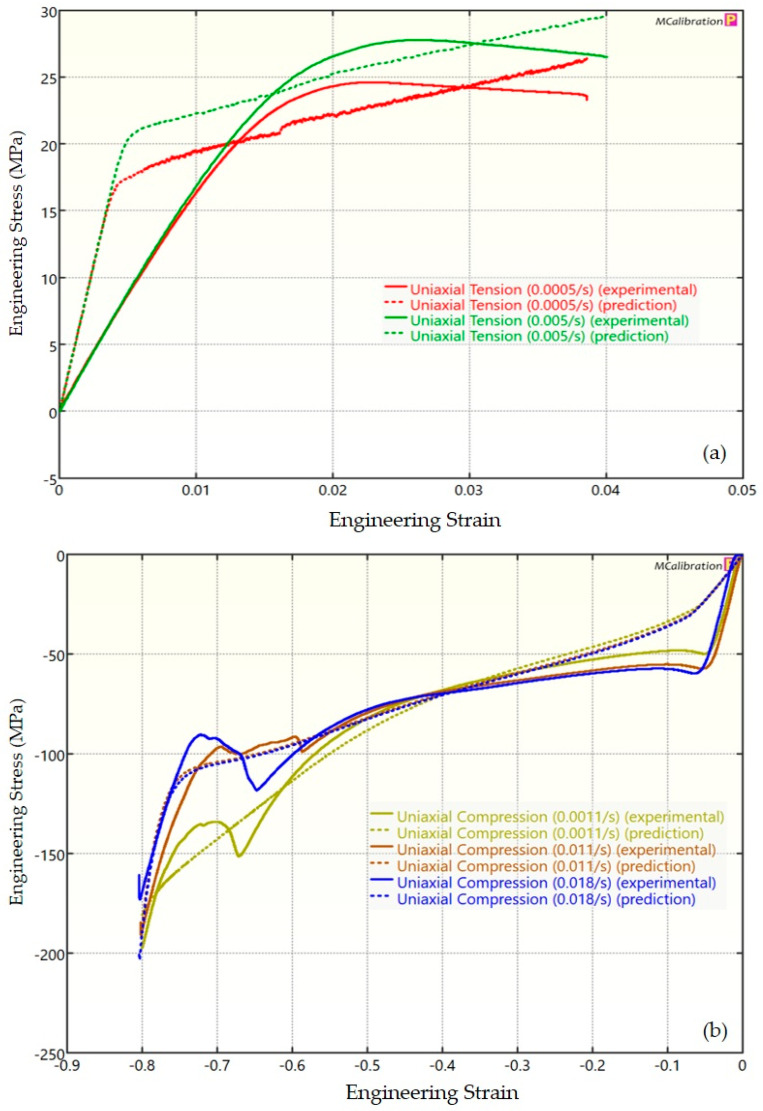
Experimental and TNV predicted results for ABS: (**a**) uniaxial tension; (**b**) uniaxial compression.

**Figure 11:**
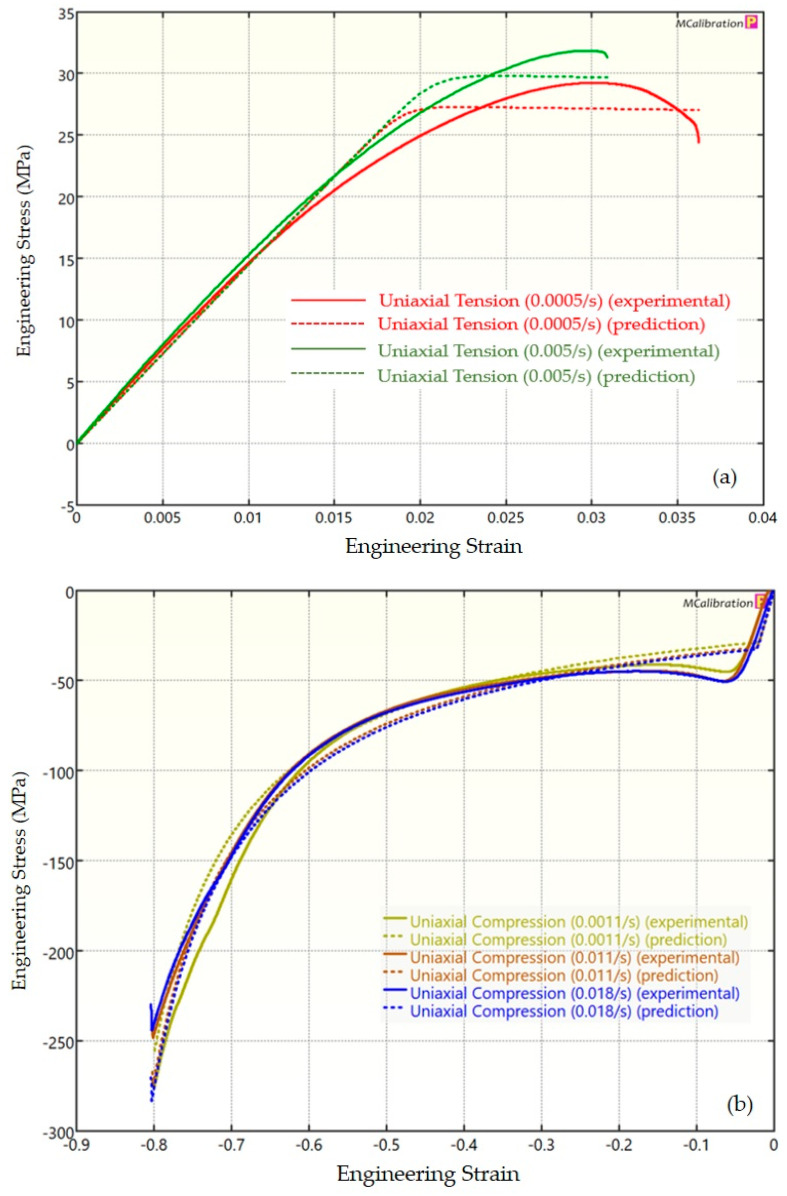
Experimental and BB predicted results for PETG: (**a**) uniaxial tension; (**b**) uniaxial compression.

**Figure 12:**
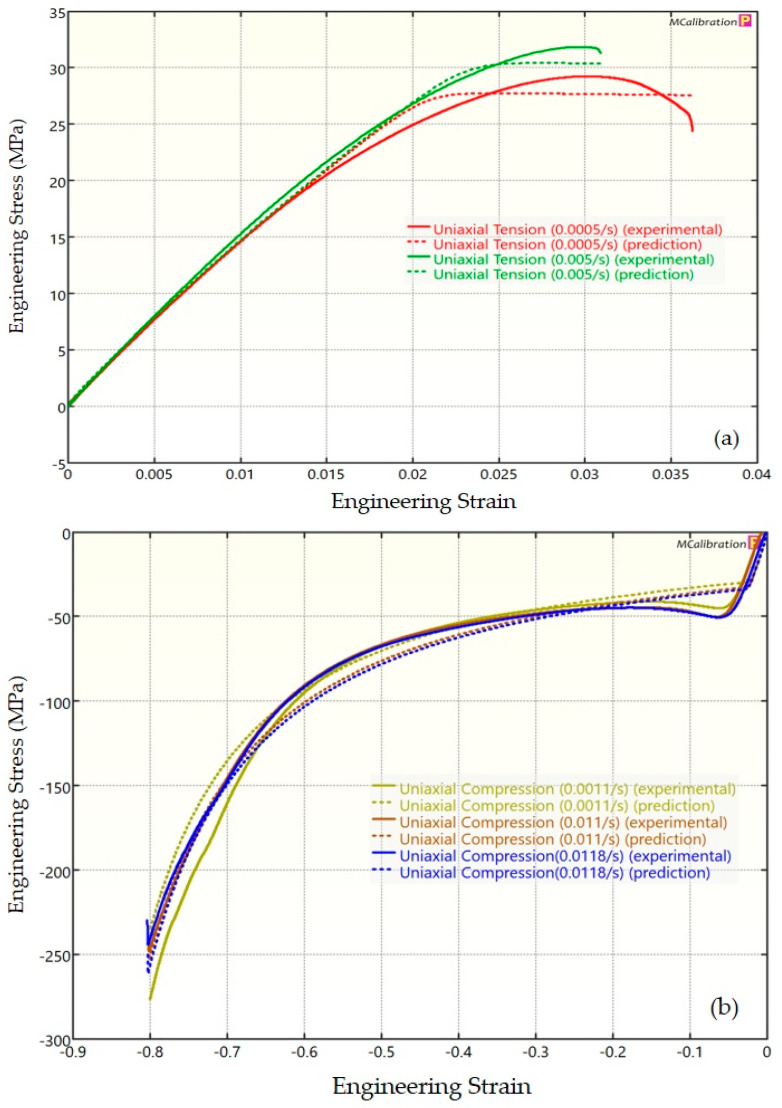
Experimental and TN predicted results for PETG: (**a**) uniaxial tension; (**b**) uniaxial compression.

**Figure 13:**
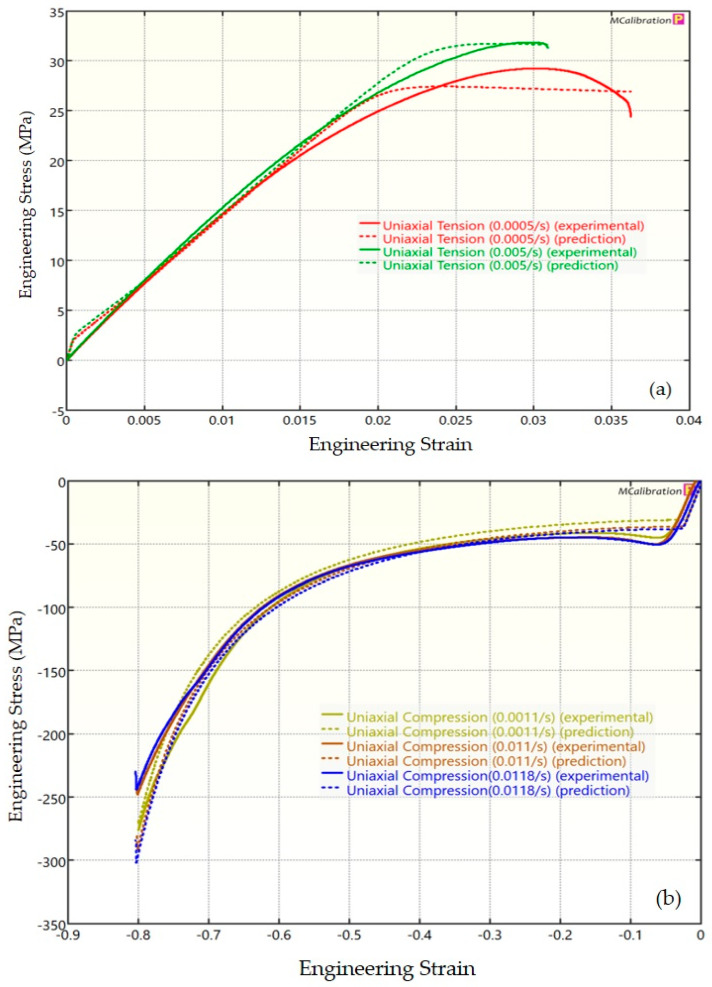
Experimental and TNV predicted results for PETG: (**a**) uniaxial tension; (**b**) uniaxial compression.

Figure 5, Figure 6, Figure 7, Figure 8, Figure 9, Figure 10, Figure 11, Figure 12 and Figure 13 present a comparison of three models for each material between experimental results and model predictions. Both the strain-rate dependency of the material and the yield evolution were reproduced by the calibrated models; this finding is consistent with those of mechanical tests. Although good agreement was observed for certain models, the same was not true for other materials. The coefficient of determination (R^2^) and average fitness error were used to quantify this. The R^2^ measures a model’s ability to explain and forecast prospective results. Its value ranges from 0 to 1, with a greater coefficient typically denoting a model with a superior fit. Moreover, the average fitness error was employed to assess the performance of different models. This indicates the accuracy of a model in making predictions. An accurate prediction model with minimal error has a low average fitness error value.

Figure 5, Figure 6 and Figure 7 present a comparison of PLA predictions made by the BB, TN, and TNV models with experimental results. These figures demonstrate how the calibrated model can accurately capture and forecast stress–strain response. For the calibrated BB model, the average fitness error was 11.588 and the coefficient of prediction (R^2^) was 0.827. Similarly, for the calibrated TN model, the R^2^ and average fitness error were 0.884 and 10.896, respectively. For the calibrated TNV model, these values were 0.886 and 12.100, respectively.

In Figure 8, Figure 9 and Figure 10, the outcomes of the calibrated BB, TN, and TNV models for ABS are displayed. For the calibrated BB model, the R^2^ was 0.756 and the average fitness error was 13.763. Likewise, the calibrated TN model had an R^2^ of 0.508 and an average fitness error of 17.028, respectively. For the calibrated TNV model, these values were 0.743 and 18.455, respectively.

Figure 11, Figure 12 and Figure 13 display the findings of calibrated BB, TN, and TNV models for PETG. For the calibrated BB model, the R^2^ and average fitness error were 0.974 and 7.343, respectively. These values were 0.973 and 6.991, respectively, for the calibrated TN model and 0.972 and 6.923 for the calibrated TNV model.

According to the R^2^ metric—which is the most favored in the literature—the best model for a given material was chosen. Furthermore, none of the models had considerably high average fitness errors. While the average fitness error is another option, the R^2^ metric was used to compare various models in this investigation. Due to its ease of interpretation and comprehension, R^2^ has been the most frequently used statistic to compare the different prediction models in the literature [50,51,52,53,54]. Table 5 lists the R^2^ values of all three models. The BB was the best material model for ABS and PETG, whereas the TNV was the best model for PLA. Therefore, to examine upper limb orthoses, the appropriate chosen model was employed for a given material. For example, the TNV material model and corresponding material constants were used to forecast deformation and stresses in design while employing PLA material for upper-limb orthoses. Likewise, the BB material model with optimal material constants was applied to predict the stresses and deformation in orthoses based on ABS and PETG. The TNV model has been chosen as the material model for the numerical analysis of the PLA-based orthosis because of its higher R^2^ value (=0.886) and comprehensiveness. This suggests that the TNV model yielded a superior fit and increased performance for PLA in comparison to the other models. In the current instance for PLA, 88.6% of the data fit the TNV model, as opposed to 82.7% and 88.4% for the BB and TN models. Generally speaking, a higher coefficient denotes a better fit for the model. Moreover, the BB model was chosen for the ABS and PETG since it yielded the highest coefficients, 0.756 and 0.974, respectively.

Each material model has its limitations and capabilities and was developed to capture certain behaviors not available with other material models. The BB model was developed for hyperelastic polymers and to incorporate large-strain dynamic behavior. TN model is more suitable for most of the thermo polymers, and it can also predict temperature-dependent phenomena, but it is unable to include failure/damage effects. In contrast, the TNV model is more general, and due to its open configuration, it can be tailored for different polymers by selecting suitable constitutive laws for each network. For example, for isothermal plastics, the following constitutive laws are recommended for each network: Network A: Yeoh hyperelascticity; Network B and C: Yeoh hyperelascticity with the power-law. With regard to the results for materials used in the study, these models are unable to accurately predict fluctuations in the strain hardening behavior of ABS during compression testing within the strain range from 0.6 to 0.8.

A brief comparison of the three material models are also introduced here for the readers’ understanding. The TN and TNV models can simulate strain rate-dependent thermal softening more accurately than the BB model owing to their ability to capture numerous deformation mechanisms [24,55]. The TNV model, which incorporates both viscoelastic and viscoplastic effects, provides the most thorough characterization and is particularly ideally suited for reproducing complicated phenomena at high strain rates [21,56,57]. Furthermore, TN and TNV models’ higher computational complexity may render them less useful for some applications than the more straightforward BB model. In comparison to the BB model, the TNV and TN models involve a significantly higher number of material constants or parameters [58]. Consequently, the model selection process should consider the specific requirements of the application as well as the required degree of prediction accuracy.

## 4. Case Study—Upper-Limb Orthosis

**Figure 14:**
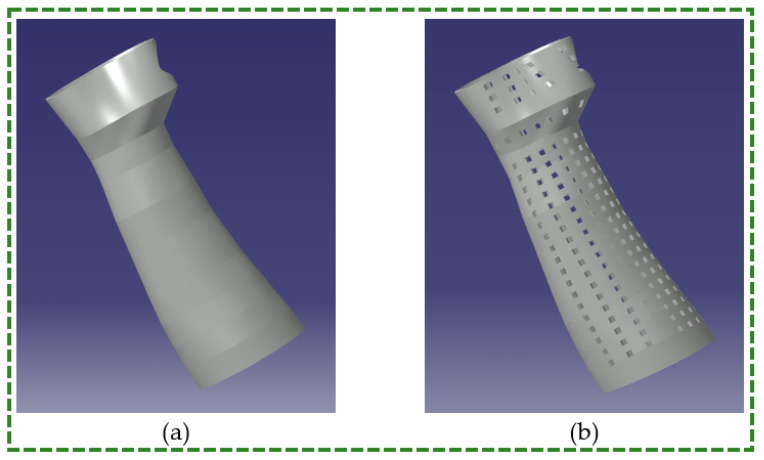
Design for hand splint (**a**) Solid (**b**) Perforated.

The upper-limb hand splint was examined in this study employing two designs, namely solid and porous (square perforated), through FE analysis. The perforated design had 22 levels with 24 perforations at a 15° angle at each level. The square perforation’s side measured 4.43 mm. Figure 14a,b depict the solid and perforated hand splint designs, respectively. The durability and dependability of the orthosis during usage are largely dependent on its strength, especially in static orthoses. However, because of the lack of suitable fixtures for their tensile or compressive testing, it is challenging to establish their strength in physical trials. Thus, a new approach was used to provide actual data using standard specimens from universal testing machines, calibrate models for different polymer materials, and then choose the most favorable model for that particular material. The material chosen was employed to assess the mechanical properties of various orthosis designs.

FE analysis was performed based on methods proposed by Cazon et al. [59]. The proposed designs comprised of 3-mm-thick solid and perforated (square-shaped) splints. For every design, ABS, PLA, and PETG were analyzed. This thickness was deemed to be suitable given the AM material’s superior stiffness. These materials’ attributes, which had been incorporated into the FE model (Table 6), were obtained through experiments and found in literature sources.

**Figure 15:**
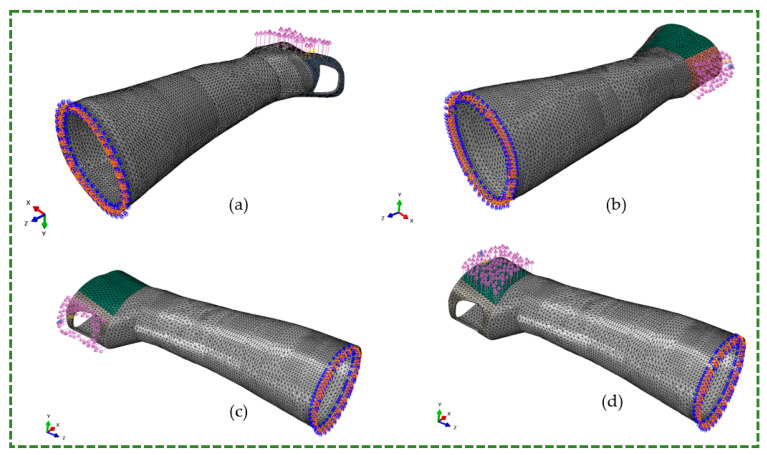
FE mesh, BCs, and load directions for four crucial wrist movements. (**a**) flexion, (**b**) extension, (**c**) radial and (**d**) ulnar.

Dassault Systems’ Abaqus software (Dassault Systems Headquarters, Vélizy-Villacoublay, France) was employed for the FE analysis of orthoses. The Initial Graphics Exchange Specification file format was used to import the designs into Abaqus. In the FE model, the splint exhibited 11,481 node points and 37,225 linear four-node tetrahedral elements (C3D4) (Figure 15).

The predominant biaxial mobility of the wrist at the carpus area results in four wrist movements: radial-ulnar deviation and flexion-extension [63,64]. The wrist’s strength required for these movements is usually determined by employing subjects who have completed static (isometric) and dynamic (isokinetic) testing. This study examined the maximum flexion-extension torque and radial-ulnar deviation torque produced by healthy volunteers [65]. Under real-world circumstances, people with stroke would often be unable to apply the complete strength necessary to break the splint. Thus, analyses at 8% of the maximal isometric strength of a healthy individual were performed. The stresses and torques imparted on the splint for these main movements are listed in Table 7.

The input force (N) required for simulation was estimated by dividing torque by the distance between the wrist and metacarpophalangeal joints [66]. Assuming that 50% of subjects (without hand-wrist medical conditions) have this measurement, the distance between the wrist and metacarpophalangeal joints was considered to be 0.1 m [67]. These force values were applied to splint surfaces that can carry these forces in real life (Table 6). It was essential to demonstrate a natural interaction between splints and hands/wrists and prevent stress resulting from point forces. The FE mesh, BCs, and load directions for the flexion, extension, radial, and ulnar are illustrated in Figure 15a–d. In addition to the most noticeable surfaces under stresses based on each movement, areas in touch with the thumb were considered for radial movement, whereas the palm section was only assumed for ulnar movement. For simulations, it was hypothesized that the patient’s forearm was at rest or fixed and the hand could be free to move but restricted by the splint. The BC could securely connect splint surfaces in the forearm region. For all four movements, the splint’s back surfaces were secured in six degrees of freedom. Each motion had a secondary limit on the central surface of the splint to establish a 3-point pressure system and matched the simulated and real-world forearm-splint relationships.

Twenty-four FE analysis simulations were conducted to examine displacements and stresses for the two designs in flexion-extension and radial-ulnar wrist movements. These simulations assessed the effectiveness of various materials and solid and perforated designs for splints. Because minor deformations were assumed in all the simulations, the materials remained in the elastic component of the stress–strain curve. The simulations were run using a static and comprehensive procedure that utilized ABAQUS/STANDARD’s implicit solver. These displacements and Von Mises stress were estimated and compared in these analyses. Every simulation was performed on a PC running Windows 10, 64 bits, with an Octa-core 3.7 GHz Xeon processor and 16 GB RAM. About one minute was needed for each simulation.

**Figure 16:**
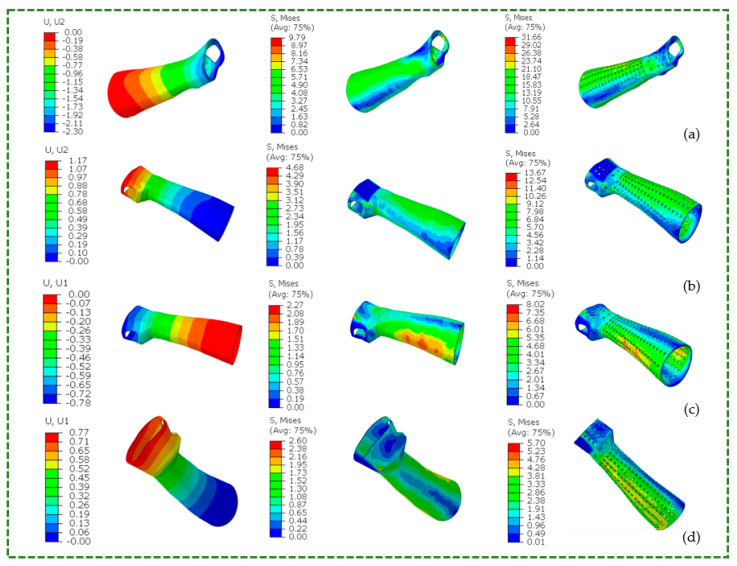
Displacement values (mm) and Von Mises stress values (MPa) for solid and perforated hand splint (**a**) flexion (**b**) extension (**c**) radial (**d**) ulnar movements in the 3-mm-thick PLA splint.

Analysis was conducted to compare different materials used in hand splint designs. The TNV model was used for PLA and the BB model for ABS and PETG. The values of displacements and Von Mises stress for the 3-mm-thick hand splint obtained from FE simulations for the 8% load are listed in Table 8 and Table 9, respectively. Figure 16 provides a graphical representation of Von Mises stress values and average displacements for PLA.

For average displacements, the PLA hand splint (both designs) was the stiffest material juxtaposed to ABS and PETG. The PLA-based solid hand splint was sturdier and more durable than the ABS-based orthosis, exhibiting approximately 11.52%, 14.61%, 12.50%, and 14.47% lower displacement in flexion, extension, radial, and ulnar directions, respectively. Likewise, the PLA-made perforated hand splint outperformed the ABS-made one, exhibiting around 18.55%, 18.60%, 17.28%, and 17.73% lower displacement in the flexion, extension, radial, and ulnar directions, respectively. The performance of PETG was poor for displacement. For example, the PETG-based solid hand splint displayed 24.69%, 21.91%, 25%, and 21.05% greater displacement than the ABS material in flexion, extension, radial, and ulnar directions, respectively. Compared with a perforated hand splint composed of ABS material, the PETG perforated hand splint demonstrated poorer performance. The PETG material exhibited approximately 17.97%, 18.18%, 18.52, and 17.33 higher displacement than ABS across flexion, extension, radial, and ulnar directions, respectively. Irrespective of these directions, splint regions with the greatest displacement were those with curvature variation, including the metacarpus. It was noted that the observed displacement was in line with the average displacement for all the materials in flexion, extension, radial, and ulnar movements. Moreover, in the perforated hand split, the overall increase in the displacements was observed for the three materials in all three directions. Undoubtedly, the existence of perforations that could serve as stress concentration sites and a smaller material volume could be the cause of the increased deformation in perforated orthosis compared to its solid variant.

Stress peaks were observed in and around the hand’s palmer digital region for the solid splint. For all the materials, the peak stress was lower than their yield strength in the solid splint (Table 6). This finding indicates that stroke patients can safely use the 3-mm-thick solid hand splint (made of PLA, ABS, and PETG). The 3-mm-thick perforated hand splint collapsed in flexion because the von Mises stress was greater than the material’s yield strength. Thus, square perforated hand splints should not be used unless significant design changes are made. Possible modifications could include thickening the splint, changing the pattern of holes, or decreasing the hole number. Moreover, the splint’s curved regions—such as its sides and the region surrounding the thumb—both on palmer and dorsal sides generate greater stress levels.

It is crucial to acknowledge that the quality of experimental data has a substantial impact on the prediction accuracy of prediction models (in this case, the BB, TN, and TNV models) [68,69,70]. 3D printing has numerous advantages, like the ability to customize, and manufacture intricate parts without the need for tools or fixtures, etc. However, proper printing conditions are needed to get the best results and reap the benefits of 3D printing. Inadequate layer adhesion, inferior dimensional accuracy, inaccurate mechanical characteristics, flawed surface quality, component imperfections, invariable part qualities, fabrication issues, etc., are possible outcomes of improper parameters selection [71,72,73,74,75,76]. The standard parts produced will be erroneous and the tensile/compression test results obtained afterward will not be reliable if the components are built using the incorrect printing parameters. Consequently, the prediction model’s calibration and the determination of material constants for a particular material will be inaccurate when based on the incorrect test results. The FE analysis of the final application (in this case, custom orthosis design) when carried out using these incorrectly calibrated material models, will produce deceptive results. Serious ramifications could arise from the possibility that a weak design could yield positive outcomes or that a well-designed orthosis could result in increased stresses or deformation. This will ultimately lead to increased expenses, material waste, user discomfort, ineffectual use of the orthosis, etc.

FE analysis and 3D printing contribute significantly to the domains of AM and biomedical engineering, especially in the development of personalized medical devices [77,78]. By using FE analysis, which can simulate different loading scenarios and other BCs, designs can be modified to meet the needs of each particular patient, increasing the medical device’s fit, appearance, comfort, and efficiency. Once analysis is completed, only 3D printing will be able to effectively build intricately shaped medical devices that mimic the bodily part. It is also feasible and economical to efficiently iterate and improve the design for user satisfaction based on their input by using FE analysis and 3D printing. This method, which uses 3D printing and FE analysis, also allows for the rapid testing a wide range of materials and designs. Moreover, with the development of customized tools based on FE models, healthcare providers can visualize the performance of different designs and materials which would be helpful in selecting the optimal design according to the patient’s specific problem and budget constraints.

Although, this study seeks to replicate the behaviors of a real patient’s hand in a splint. However, this research overlooked or oversimplified a number of dynamics because there was not enough information available. It is challenging to identify the appropriate load intensity because wrist joints with stroke are not yet well-studied. Due to this lack of information, it was assumed that strength loss in a wrist impaired by stroke is comparable to that in a wrist affected by arthritis. Future research should incorporate the actual pressure that a stroke patient might consciously apply to a splint in a true deployment scenario. Nonetheless, this study used real experimental data to obtain the mechanical parameters needed for FE simulations. Because these data were gathered in actual settings, they may fairly depict real-world circumstances. Other materials with higher elasticity and solutions to thicken the splint and incorporate unique lattice architecture should be examined. The effects of other loads and BCs on FE simulations should also be considered.

## 5. Conclusions

To assess a material’s suitability for any given application, its mechanical behavior, especially that of polymeric materials, should be examined. Standard specimens must be used for tensile and compression testing when using a universal testing machine, which makes physical testing resource-intensive. To perform mechanical testing for orthosis, a particular fixture must be designed; this is an expensive and time-consuming process. Thus, FE analysis is utilized to identify the mechanical behaviors of an upper-limb hand orthosis with a suitable material model.

Three constitutive mathematical models—BB, TN, and TNV for PLA, ABS, and PETG—were investigated. The optimal model for a material was identified based on R^2^, having first been calibrated using data from physical tests. Appropriate material parameters, which were established using various optimization techniques, were necessary for the calibration of material models. This investigation revealed that TNV was the most appropriate model for PLA, whereas BB was the best model for ABS and PETG. Subsequently, the hand orthosis based on two designs and three materials was examined, each using its material model. The PLA hand splint (both designs) had the lowest average displacement, followed by ABS and PETG. Additionally, compared with solid hand splints, perforated splints exhibited overall increase in displacements in flexion, extension, radial, and ulnar directions for all three materials. In the solid splint, the peak stress was lower than yield strength. These findings indicate that stroke patients can safely use the 3-mm-thick solid hand splint. However, the 3-mm-thick perforated hand splint failed in flexion because the von Mises stress was greater than the material’s yield strength. Further design exploration would be advantageous, even though this would require volunteers and financial resources. The cost-effectiveness and utility of diverse materials for 3D-printed splints will eventually need to be demonstrated through human research.

This work has used FE simulation to investigate the mechanical performance of the orthosis designs under different loading scenarios. Despite the fact that FE modeling is an invaluable tool, it is unable to accurately represent the complex and dynamic mechanical stresses that arise in real-world applications [79]. Thus, real-world clinical evaluation is required in addition to FE simulation to validate the effectiveness of orthoses composed of different materials. Similarly, FE simulation cannot be used to assess comfort, which is a subjective performance indicator. Characterization of comfort necessitates more research, such as clinical tests, user feedback, ergonomic assessment, etc., since it depends on fit, skin response, aesthetics, and other factors. Although FE models can provide valuable insights into the mechanical characteristics of orthosis designs. Yet, real-world clinical efficacy is still required, especially with regard to comfort, durability, and therapeutic efficacy. It is undoubtedly possible to achieve enhanced upper limb rehabilitation for increased patient satisfaction through optimal orthosis design by combining FE simulation with clinical assessments. The clinical assessment is not included in this work due to resource constraints; however, clinical trials will be conducted in the future to further improve the design and enhance the reliability of the findings. Furthermore, the future direction regarding material modeling is to develop more accurate and robust material models that can handle multiple scenarios that could occur during the use of 3D-printed orthotic devices. Another area is to develop and calibrate material models with temperature dependency for the simulation of distortion and residual stress development during 3D printing. FE models for simulating 3D printing of polymers for orthotic devices could save a lot of time and cost usually associated with trial experimental runs.

## Figures and Tables

**Table 1:** Three-dimensional printing parameters utilized for producing standard specimens.

Material	Variables					
	Bed Temperature (°C)	Nozzle Temperature (°C)	Speed (mm/s)	Layer Thickness (mm)	Infill (%)	Extrusion Width (mm)
PLA	55	205	70	0.1	100	0.4
ABS	100	250				
PETG	60	245				

**Table 2:** Material parameters used by the Bergström–Boyce.

Symbol	Value	Description
μ	2.11919	Shear modulus of network A
*λ_L_*	6.27995	Locking stretch
*κ*	42616.8	Bulk modulus
*s*	340.526	Relative stiffness of network B
*x_i_*	0.83272	Strain adjustment factor
c	−0.0189804	Strain exponential
*τ*	32.418	Flow Resistance
*m*	24.999	Strain exponential
*τ_cut_*	0.01	Normalized cut-off stress for flow

**Table 3:** Material parameters used by the three-network model.

Symbol	Value	Description
$\mu_{A}$	355.991	Shear modulus of network *A*
*λ_L_*	4.21835	Locking stretch
*κ*	4261.68	Bulk modulus
$\hat{\tau_{A}}$	12.4453	Flow resistance of network *A*
*a*	0.00596287	Pressure dependence of flow
*m_A_ = m_B_*	22.2823	Stress exponential of network *A*
*μ_Bi_*	395.969	Initial shear modulus of network *B*
*μ_Bf_*	273.751	Final shear modulus of network *B*
*β*	9.42152	$\mathrm{Evolution}\;\mathrm{rate}\;\mathrm{of}\;\mu_{B}$
$\hat{\tau_{B}}$	21.5989	Flow resistance of network B
$\mu_{C}$	0.805924	Shear modulus of network C
*q*	0	Relative contribution of I_2_ of network C

**Table 4:** Material parameters used by the Three Network Viscoplastic model.

Symbol	Value	Description
C10	3.34032	Yeoh parameter 1 of Network A
C20	−0.210461	Yeoh parameter 2 of Network A
C30	0.00479607	Yeoh parameter 3 of Network A
*κ*_1_	2130.84	Bulk modulus 1 of Network A
*κ*_2_	0	Bulk modulus 2 of Network A
*κ*_3_	0	Bulk modulus 3 of Network A
C10	596.03	Yeoh parameter 1 of Network B
C20	0	Yeoh parameter 2 of Network B
C30	0	Yeoh parameter 3 of Network B
*κ*_1_	2130.84	Bulk modulus 1 of Network B
*κ*_2_	0	Bulk modulus 2 of Network B
*κ*_3_	0	Bulk modulus 3 of Network B
$\hat{\tau_{B}}$	9.93545	Flow resistance of network B
mm	19.6805	Stress exponent of Network B
bb	0	Volumetric flow coefficient of Network B
p_o_	−0.194728	Pressure dependence of flow of Network B
fff	1.40761	$\mathrm{Yield}\;\mathrm{evaluation}\;\mathrm{of}\;\hat{\tau_{B}}$
epsF	0.489528	Yield evaluation of characteristics strain of Network B
ceps	0.1	Flow damage strain of Network B
fss	1	Flow damage final state of Network B
C10	239.134	Yeoh parameter 1 of Network C
C20	0	Yeoh parameter 2 of Network C
C30	0	Yeoh parameter 3 of Network C
*κ*_1_	2130.84	Bulk modulus 1 of Network C
*κ*_2_	0	Bulk modulus 2 of Network C
*κ*_3_	0	Bulk modulus 3 of Network C
$\hat{\tau_{C}}$	51.8926	Flow resistance of network C
mm	8.59769	Stress exponent of Network C
bb	0	Volumetric flow coefficient of Network C
p_o_	0.77947	Pressure dependence of flow of Network C
fff	0.25083	$\mathrm{Yield}\;\mathrm{evaluation}\;\mathrm{of}\;\hat{\tau_{c}}$ of Network C
epsF	0.236314	Yield evaluation of characteristics strain of Network C
ceps	0.1	Flow damage strain of Network C
fss	1	Flow damage final state of Network C

**Table 5:** Summary of R^2^ values for different material models.

Material	Material Model		
	BB	TN	TNV
PLA	0.827	0.884	0.886
ABS	0.756	0.508	0.743
PETG	0.974	0.973	0.972

**Table 6:** Properties of the 3D printing materials.

Material	Properties		
	Young’s Modulus (GPa)—Estimated	Poisson Ratio	Yield Strength (MPa)—Estimated
PLA	2.28	0.30 [60]	25.98
ABS	1.80	0.35 [61]	20.70
PETG	1.64	0.33 [62]	19.55

**Table 7:** Considered torque and loads applied to the splints.

	Vanwearingen Torque (N m)	Applied 8% Loads (N)	Load Direction
Flexion	14.8	11.8	−Y-axis
Extension	8.4	6.7	Y-axis
Radial deviators	11.4	9.1	−X-axis
Ulnar deviators	9.9	7.9	X-axis

**Table 8:** Highest displacement (mm) attained in the major axes for 8% load.

Design	Material		Flexion (−Y) (mm)	Extension (Y) (mm)	Radial Deviators (−X) (mm)	Ulnar Deviators (X) (mm)
Solid	PLA	X	−0.25	0.09	−0.78	0.77
		Y	−2.30	1.17	−0.07	0.13
		Z	0.40	0.26	0.29	−0.25
	ABS	X	−0.22	0.08	−0.93	0.93
		Y	−2.68	1.40	−0.05	0.12
		Z	0.47	0.30	0.34	−0.29
	PETG	X	−0.32	0.12	−1.12	1.11
		Y	−3.28	1.68	−0.09	0.17
		Z	0.57	0.37	0.41	−0.36
Perforated (Square Perforations)	PLA	X	−0.20	0.08	−0.95	0.73
		Y	−3.38	1.56	−0.07	0.12
		Z	−0.59	0.33	0.35	−0.23
	ABS	X	−0.23	0.10	−1.18	0.91
		Y	−4.14	1.92	−0.07	0.13
		Z	−0.75	0.40	0.44	−0.29
	PETG	X	−0.28	0.12	−1.38	1.06
		Y	−4.90	2.26	−0.09	0.16
		Z	−0.86	0.48	0.51	−0.34

**Table 9:** Von Mises stress values (MPa) achieved for 8% load.

Design	Material	Flexors (−Y) (MPa)	Extensors (Y) (MPa)	Radial Deviators (−X) (MPa)	Ulnar Deviators (X) (MPa)
Solid	PLA	9.79	4.68	2.27	2.60
	ABS	5.74	2.71	2.08	2.06
	PETG	8.16	3.61	2.10	2.22
Perforated (Square (Perforations)	PLA	31.66	13.67	8.02	5.70
	ABS	33.05	15.10	9.25	6.55
	PETG	36.61	15.48	8.51	6.03

## Data Availability

The data presented in this study are available in the article.

## Nomenclature

Symbol	Description
μ	Shear modulus of network A
*λ_L_*	Locking Stretch
*κ*	Bulk Modulus
*s*	Relative stiffness of network B
*x_i_*	Strain adjustment factor
c	Strain exponential
*τ*	Flow Resistance
*m*	Strain exponential
*τ_cut_*	Normalized cut-off stress for flow
$\lambda_{B}^{e*}$	Chain stretch in the elastic portion of Network B
*σ*	Cauchy stress
*L_B_*	Velocity gradient on network B
$\tilde{D_{B}^{v}}$	Viscous deformation rate of Network B
$\hat{\tau_{cut}}$	Cutoff stress
$\mu_{A}$	Shear modulus of network *A*
*λ_L_*	Locking stretch
*κ*	Bulk modulus
$\hat{\tau_{A}}$	Flow resistance of network *A*
*a*	Pressure dependence of flow
*m_A_* = *m_B_*	Stress exponential of network *A*
*μ_Bi_*	Initial shear modulus of network *B*
*μ_Bf_*	Final shear modulus of network *B*
*β*	$\mathrm{Evolution}\;\mathrm{rate}\;\mathrm{of}\;\mu_{B}$
$\hat{\tau_{B}}$	Flow resistance of network B
$\mu_{C}$	Shear modulus of network C
*q*	Relative contribution of I_2_ of network C
θ	Current temperature
θ_0_	Reference temperature
$\hat{\theta }$	Stiffness’s temperature dependence
$L^{-1}$ (*x*)	Inverse Langevin function
*κ*	Bulk modulus
$\dot{Y_{A}}$	Viscoplastic flow rate
$p_{A}$	Hydrostatic pressure
*R*(*x*)	Ramp function
